# Algorithmic transparency and interpretability measures improve radiologists’ performance in BI-RADS 4 classification

**DOI:** 10.1007/s00330-022-09165-9

**Published:** 2022-10-25

**Authors:** Friederike Jungmann, Sebastian Ziegelmayer, Fabian K. Lohoefer, Stephan Metz, Christina Müller-Leisse, Maximilian Englmaier, Marcus R. Makowski, Georgios A. Kaissis, Rickmer F. Braren

**Affiliations:** 1 Institute of Diagnostic and Interventional Radiology, School of Medicine, Technical University of Munich, 81675 Munich, Germany; 2 Department of Computing, Faculty of Engineering, Imperial College of Science, Technology and Medicine, London, SW7 2AZ UK; 3 Institute for Artificial Intelligence in Medicine and Healthcare, School of Medicine and Faculty of Informatics, Technical University of Munich, 81675 Munich, Germany; 4 German Cancer Consortium (DKTK) Partner Site Munich, 69120 Heidelberg, Germany

**Keywords:** Artificial intelligence, Trust, Algorithms, Radiologists, Perception

## Abstract

**Objective:**

To evaluate the perception of different types of AI-based assistance and the interaction of radiologists with the algorithm’s predictions and certainty measures.

**Methods:**

In this retrospective observer study, four radiologists were asked to classify Breast Imaging-Reporting and Data System 4 (BI-RADS4) lesions (*n* = 101 benign, *n* = 99 malignant). The effect of different types of AI-based assistance (occlusion-based interpretability map, classification, and certainty) on the radiologists’ performance (sensitivity, specificity, questionnaire) were measured. The influence of the Big Five personality traits was analyzed using the Pearson correlation.

**Results:**

Diagnostic accuracy was significantly improved by AI-based assistance (an increase of 2.8% ± 2.3%, 95 %-CI 1.5 to 4.0 %, *p* = 0.045) and trust in the algorithm was generated primarily by the certainty of the prediction (100% of participants). Different human-AI interactions were observed ranging from nearly no interaction to humanization of the algorithm. High scores in *neuroticism* were correlated with higher persuasibility (Pearson’s *r* = 0.98, *p* = 0.02), while higher *consciousness* and change of accuracy showed an inverse correlation (Pearson’s *r* = −0.96, *p* = 0.04).

**Conclusion:**

Trust in the algorithm’s performance was mostly dependent on the certainty of the predictions in combination with a plausible heatmap. Human-AI interaction varied widely and was influenced by personality traits.

**Key Points:**

*• AI-based assistance significantly improved the diagnostic accuracy of radiologists in classifying BI-RADS 4 mammography lesions.*

*• Trust in the algorithm’s performance was mostly dependent on the certainty of the prediction in combination with a reasonable heatmap.*

*• Personality traits seem to influence human-AI collaboration. Radiologists with specific personality traits were more likely to change their classification according to the algorithm’s prediction than others.*

**Supplementary Information:**

The online version contains supplementary material available at 10.1007/s00330-022-09165-9.

## Introduction

Artificial intelligence (AI) has emerged as a promising diagnostic tool in medical applications. Recent studies in medical imaging have demonstrated performance of AI systems equal or superior to human readers, for example in mammography-based breast cancer screening [1–3]. These results encourage the integration of deep learning–based computer-aided diagnosis (CAD) into clinical and radiological practice. However, substantial technical obstacles, including poor generalization of trained algorithms [4] and difficulties in workflow integration [5], hinder broad introduction of such systems into the daily routine. Additionally, human factors including skepticism against the use of algorithms [6], or lacking trust in algorithm-based predictions [7] can hinder algorithm usage by clinicians. A successful introduction into clinical practice must thus overcome several obstacles and consider human-AI interaction [8].

Investigation into the impact of human-AI interaction on the efficacy of applying algorithmic predictions to clinical decision-making and on clinical outcome metrics (like diagnostic or predictive accuracy) is fundamental for the clinical translation of AI systems. Early studies investigating various prompt types for CAD systems have shown a significant influence of different displays of computer-based assistance on the performance of radiologists as well as on their attention towards different image areas [9–12]. However, recent studies investigating the influence of more advanced, AI-based clinical support systems have concentrated to show on-par or even superior performance of algorithms in various medical imaging classification tasks [13–15]. Nevertheless, human perception of and interaction with those algorithms still awaits deeper investigation. First studies have shown an influence of personality traits on the human-AI interaction and trust in the algorithm [16, 17]. Trustworthy machine learning, which is often defined as explainable, fair, verifiable, transparent, and robust [18], is crucial for successful human-AI collaboration [19, 20]. Quantifying the uncertainty of predictions to inform users about the reliability of the model’s outputs as well as a visual display of image regions of high importance for the AI-based prediction are two methods to create transparent, interpretable, and trustworthy AI systems [21, 22]. Especially in radiological settings, this offers the possibility of expert-based quality control and allows for enhanced image interpretation, which can consequently increase the trust in the algorithm.

In this study, we trained a 50-layer residual neural network (ResNet50) on an open database (CBIS-DDSM) [23] consisting of 1696 Breast Imaging-Reporting and Data System (BI-RADS) mammograms to classify benign and malignant lesions. We subsequently tested the algorithm on an independent in-house test set of 200 BI-RADS 4-labeled lesions identified on mammograms with an additional label of “benign” (*n* = 101) or “malignant” (*n* = 99) from histopathology-derived ground-truth. We then independently analyzed the influence of algorithm-derived classification, certainty, and attention map on the performance of radiologists with varying levels of experience in classifying the independent test set in benign and malignant lesions. Furthermore, the human-AI interaction and the radiologists’ perception of the algorithm were observed.

## Material and methods

### Network training and data

We fine-tuned a ResNet50 with weights pretrained on ImageNet [24] to classify lesions on 1696 out of 2620 mammograms from the Curated Breast Imaging Subset of the Digital Database for Screening Mammography (CBIS-DDSM) [23] by first training the fully connected layers of the network only, followed by successive unfreezing of earlier layers, using the TensorFlow framework [25]. The images contained BI-RADS 4 lesions with respective histopathological ground truth (malignant, benign). Data augmentation was applied by rotating up to 90°, flipping, shifting by 10%, or zooming in up to 20%. The data was divided into a training and validation set using a 80%/20% random split. The ResNet50 was trained using a batch size of 64 images and binary cross-entropy as loss. The initial learning rate was 0.0001 with a triangular learning policy and halving in case of no decrease over two epochs. Nesterov-accelerated adaptive moment estimation (Nadam) [26] was used as optimizer and early stopping was applied after five epochs without an improvement in validation loss. We trained to convergence and validation loss was used for final model selection.

### Independent test set

A set of 200 age and breast density–matched full-field digital mammograms (CC and MLO views) obtained between February 2009 and February 2018 were retrospectively acquired from our institution for both network validation and usage in the reader study. All mammograms contained one mass each classified as BI-RADS 4 and all patients underwent biopsy within one month after the diagnostic mammogram. Histopathological labels served as ground truth (*n* = 101 benign, *n* = 99 malignant). All images were preprocessed by placing a bounding box around each lesion and adjusted to the size of the training set to match the training data. By doing so, the cropped images simulate the output of an object detection algorithm applied to the mammogram, as common in AI applications.

### AI-based assistance

**Fig. 1:**
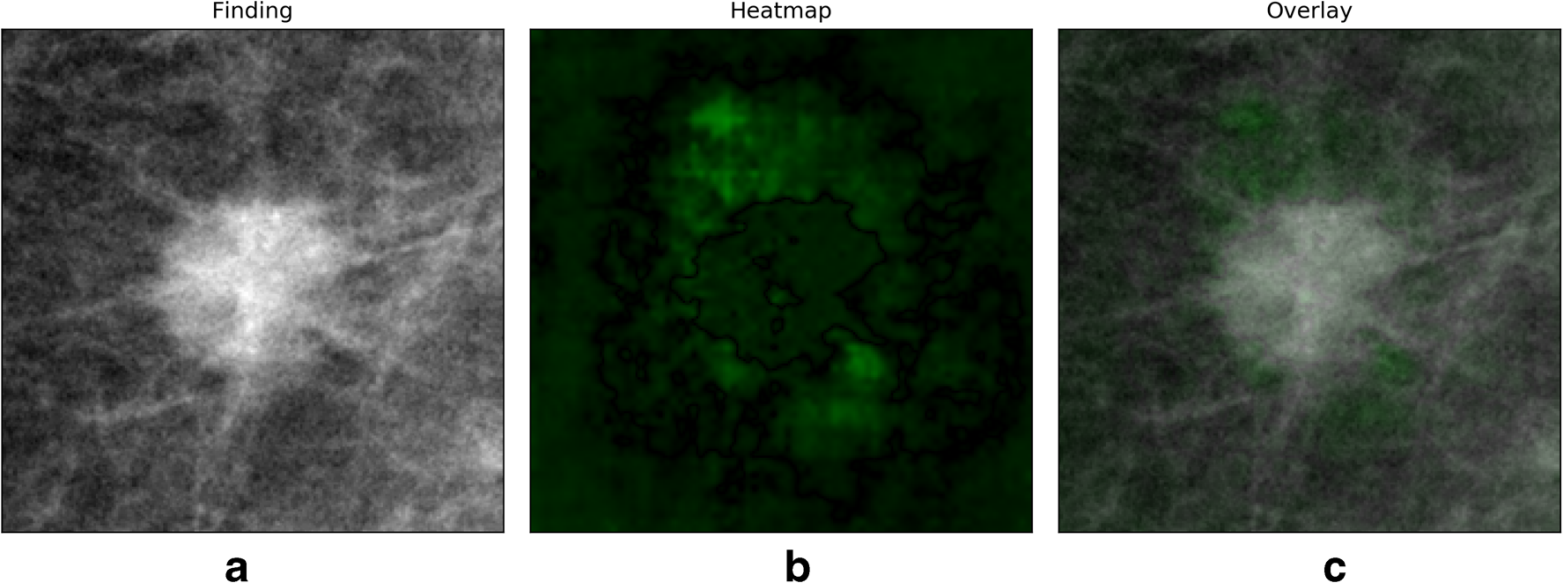
Heatmap assistance on the example of a malignant lesion: (**a**) Mammogram, (**b**) heatmap, and (**c**) overlay

Different methods of AI-based assistance were tested. A heatmap highlighting the image’s most important areas for classification was created by computing the occlusion-based sensitivity map for each image [27]. Here, pixels were colorized according to their contribution to the prediction. Heatmaps were displayed on their own or overlayed with the cropped image from the original mammogram, as displayed in Fig. 1.

Furthermore, the calculated certainty of the ResNet50’s prediction using the classification scores was displayed. As the study excluded out-of-sample images, the certainty of the prediction was estimated by calculating the probability for the class “malignant” and displayed thus or as 1-probability for benign classifications. In the study, a display of the probability of the predicted class ranging between 0 and 100% was added to the heatmap presentation.

### Design of reader study

Four radiologists from our institute were recruited for the study which took place between June and September 2020. For the study, diagnostic monitors with a resolution of 2048 × 2560 pixels calibrated to the DICOM GDFS were used and the ambient light was set below 50 lx. None of the participants had evaluated the mammograms used for the study during the last 27 months. To minimize decision bias from additional information sources, the radiologists were not given any clinical meta-information about the patients. The radiologists represented different stages of proficiency (one attending with a focus on mammography (S.M., 20 years of experience, > 3000 mammograms per year), one attending with a focus on abdominal imaging (F.L., 7 years of experience, ca. 500 mammograms per year), one consultant radiologist (M.E., 6 years of experience, > 1000 mammograms per year), and one radiology resident (C.M-L., 2,5 years of experience, ca. 500 mammograms per year)).

At first, the readers were asked to classify the cropped images simulating the output of an object detection algorithm applied to the mammograms of all 200 images of the test set into benign or malignant findings. They were given no additional clinical data but were told, that all images contained lesions classified as BI-RADS 4 before. In a second step after 6 weeks, the readers were asked to classify the images again to assess intra-reader reproducibility. Directly afterwards, the radiologists were incrementally and additively provided with the different types of output display generated by the ResNet50 (heatmap, prediction, and certainty), and human-AI interaction was observed using a questionnaire and by an observing person during the session of the study. After each output presentation, the readers could either adhere to their original classification or change it.

After performing the classification task, each reader was interviewed in a structured fashion and answered a questionnaire regarding their radiological experience and attitude towards AI applications in the medical field, which is attached in the supplement material. We analyzed the possible influence of participants’ personalities on human-AI interaction and the resulting performance using a publicly available personality test [28] based upon the Big-Five personality model [29–31] and items from the International Personality Item Pool [32]. As these personality traits were found to be mostly stable in adults [33], we chose the model as being presumably independent from working experience and age.

### Statistics

Statistical analyses were performed using Python 3.8.2. A two-sided significance level of *α* = 0.05 was chosen for all tests. For comparison of the radiologists’ performance with and without AI assistance, the arithmetic mean over the readers’ accuracy on each task was calculated and McNemar’s test with Yates correction was calculated based on the classification changes made by the radiologists. In concordance with the International Personality Item Pool recommendation, analysis of personality traits and their inference with human-AI-interaction using Pearson’s correlation was performed based on the absolute scores in each Big-Five personality model [29] category.

## Results

The STROBE checklist [34] and patient inclusion flowchart for the in-house test set can be found in the supplement material.

### Model performance and human reader setting

The ResNet50 achieved a sensitivity of 76%, specificity of 70% , and ROC-AUC of 0.80 on the test set. The radiologists’ accuracy on classifying the cropped mammograms without any AI assistance was 75.0% ± 1.6%. With no clinical data provided, 50% of the participants experienced the classification task more difficult than in their clinical routine while the rest considered it equivalent. Cohen’s kappa for the two assessments ranged between 0.31 and 0.60, revealing a fair to moderate intra-reader agreement, with the two attendings having higher values than the two less experienced readers.

### Human-AI collaboration

#### Influence on human performance

Overall, algorithm-based assistance consisting of heatmap, classification, and network’s certainty led to a significant improvement in the diagnostic accuracy from 75.0% ± 1.6% to 77.8% ± 1.2% (increase of 2.8% ± 2.3%, 95 %-CI 1.5% to 4.0%, two-sided *t*-test: *p* = 0.045). In contrast, no significant improvement was observed when providing the heatmap only. Changes based solely upon heatmaps had a higher risk of being false (55% versus 37%). Table 1 displays the mean performance achieved by the radiologists within the different steps of the study.

**Table 1:** Reader’s performance with different ResNet50 assistance

AI based assistance	Sensitivity (%)	Specificity (%)	Accuracy (%)	*p* value (∆ accuracy)
No AI support	75.5 ± 5.2	74.8 ± 5.2	75.0 ± 1.8	--
Heatmap	76.3 ± 6.2	76.7 ± 6.4	75.3 ± 1.6	0.839
Heatmap + Prediction + Certainty	79.3 ± 6.7	76.2 ± 8.7	77.8 ± 1.3	0.045

#### Classification changes

**Fig. 2:**
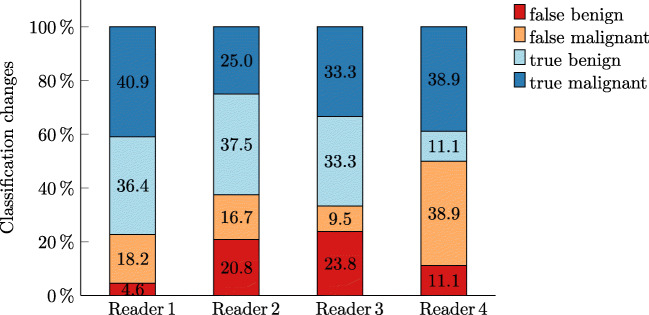
Percentual classification changes by each reader: Cumulated overall study stages (with the assistance of heatmap alone and with additional certainty and prediction) and divided into subgroups of right or false changes to benign or malignant findings. Reader 1: Resident, Reader 2: Consulting, Reader 3: attending with a focus on mammography, Reader 4: attending with a focus on abdominal radiology

Based on the algorithmic assistance, the participants changed their original classification on average 21.3 ± 2.5 times (5.3 %  ± 0.63) and in the majority (64.2%) this change resulted in the correct diagnosis. In addition, only 15.1% (95%-CI: 10.8 to 19.3%) of all changes resulted in a false negative (i.e., benign) classification while 34.5% (95%-CI: 31.2 to 37.9%) led to true positive (i.e., malignant) classification. This resulted in a statistically significant increase in the diagnostic performance of radiologists with and without AI assistance (McNemar’s test: *χ*^2^ = 7.1, *p* = 0.009). An overview of the percentual changes made by each reader can be seen in Fig. 2.

### Questionnaire-based evaluation of AI assistance

The evaluation and perception of the algorithm’s assistance were evaluated using a questionnaire, which is provided in the supplement material (A3).

#### Heatmap

The heatmap, although not significantly increasing the diagnostic accuracy on its own, was valued by the radiologist as a useful tool for quality control of the algorithm (3/4 radiologists) and allowed a comparison between their own visual findings and regional importance (2/4 radiologists). Especially experienced (i.e., attending-level) radiologists valued this aspect of possible quality and plausibility control leading to increased confidence in the algorithm.

#### Certainty of prediction

Half of the participants considered the display of the model’s prediction as most important for their final classification, whereas the other half considered the certainty of the prediction to be more relevant. However, all radiologists stated that both the prediction of the ResNet50 and its certainty were relevant for their trust in the validity of the prediction and consequently influenced their willingness to change their classification. The radiologists reported that certainty of 70–80% of the algorithm’s prediction in combination with a plausible heatmap caused them to revise their initial decision.

### Attitude towards AI integration into clinical workflow

#### Radiological experience

On average, the radiologists had 9.4 years of experience in radiology, thereof 3.3 years of analyzing mammograms. All radiologists stated to ask for advice from experienced colleagues in difficult cases with the less experienced radiologists more often than the experienced ones. Furthermore, more experienced radiologists tended to reach a final decision regarding the radiological report in clinical routine with the first read, while less experienced colleagues often reassessed the report in the short term for a final record (Pearson’s *r* = 0.97, *p* = 0.03).

#### Possible aid of the ResNet50

Three out of four radiologists stated that the AI support helped them in identifying both benign and malignant findings, while one radiologist saw the main aid of the AI in identifying malignant findings. This aligns with the increase of both sensitivity and specificity of the participants as shown in Table 1. However, only half of the radiologists viewed their performance to be improved with the assistance of the algorithm.

#### Attitude towards AI in clinical setting

All radiologists considered the impact of integrating AI into clinical workflows mainly positive. In future practice, three out of four expect time savings with AI involvement, and half expect an increase in diagnostic accuracy. However, half also foresee more carelessness when integrating AI systems. Even so, none of the participating radiologists expect a drop in radiologists’ performance when using AI tools in clinical practice and all see the integration as a second reader as a possible application. The potential benefit expected from applying the algorithm varied among radiologists. Half believed it could only help in simple cases, whereas one clinician also expected support for complicated cases. Only one radiologist stated that they do not expect AI to be of any help for their future practice.

### Human-AI interaction

#### Communication and perception of cooperation

Three of the four radiologists felt they cooperated with the algorithm in classifying mammograms rather than competing with it. Furthermore, three of them felt relieved, when the algorithm classified the images into the same category as they did and half of them felt increased stress levels if the prediction did not meet their finding. The perception was unrelated to the clinical experience of the radiologists. “Communication” with the algorithm varied between the participants ranging from no interaction to treating the algorithm as they would a human. In particular, one radiologist talked to the computer, praised the algorithm when he believed the prediction to be correct or identified an important aspect leading to a classification change (“great,” “well done”), and rebuked it when predictions diverged (“we really disagree on this one”).

#### Influence of personality traits

Radiologists with higher values in *neuroticism* trait tended to change their original classification more often when working with the algorithm (Pearson’s *r* = 0.98). Participants with higher scores in *extraversion* profited more from the AI assistance (Pearson’s *r* = 0.96). Furthermore, high scores in *consciousness* were negatively correlated with a change in accuracy (Pearson’s *r* = −0.96). *Agreeableness* and *openness* showed no correlation with the number of changes made or a change in accuracy. Table 2 depicts the Pearson correlation coefficient between personality traits and reader performance.

**Table 2:** Pearson correlation coefficient between personality traits and readers performance. Correlation between accuracy change and number of changes with personality trait scores achieved in the Big-Five model

Personality trait	∆ accuracy	Number of changes
Agreeableness	−0.74	−0.03
Consciousness	−0.96	−0.74
Extraversion	0.96	0.71
Neuroticism	0.82	0.98
Openness	0.34	−0.18

## Discussion

We show that the diagnostic accuracy of radiologist’s performance on BI-RADS 4 classification is significantly improved by combined AI-based assistance consisting of an attention heatmap and algorithm certainty indication (2.8% ± 2.3%, 95 %-CI 1.5 to 4.0 %, *p* = 0.045). The willingness to change a diagnosis upon AI-based assistance was most dependent on the AI certainty level (all participants). Furthermore, high *neuroticism* scores correlated with the number of changes made upon AI-based assistance (Pearson’s *r* = 0.98, *p* = 0.02), while *consciousness* showed an inverse correlation (Pearson’s *r* = −0.96, *p* = 0.04). The diagnostic performance of readers increased using AI-based assistance and all participants stated that AI could act as a second reader in the possible future radiological workflow. In concordance with previous literature [2, 35, 36], our study thus supports AI as a second reader in radiological settings.

AI applications require the use of interpretability and explainability tools to generate trust and bridge the gap in clinical workflow integration [18, 19]. In our study, the sole heatmap did not improve diagnostic accuracy and was deemed least useful. The radiologists stated that the visualization was unintuitive initially, as model activations were primarily located in the transition zone between tumor and surrounding parenchymal or fatty tissue outlining the lesion. In contrast, radiologists focused on lesion-specific aspects like asymmetry or architectural distortion. However, all participants stated that they became “more comfortable” with the heatmap’s appearance during the study, which was ultimately deemed a good measure of the algorithm’s prediction quality, indicating a positive effect on transparency and understandability. This aligns with similar research, where lack of validation possibilities inhibited the usage of CAD assistance [37] and that AI-based assistance in accordance with the given task improves the diagnostic accuracy of readers more than other types of AI support [38].

The readers in our study displayed a mainly positive attitude towards AI in medical settings, contradicting recent reports of hidden anti-algorithm skepticism [6]. This may represent a source of bias, as radiologists with different attitudes towards the AI-based assistance may benefit differently.

Considering personality traits, higher scores in *neuroticism*, the tendency to experience negative emotions [39], correlated strongly with the number of changes made by readers during the study. This is in contrast to recent findings of neuroticism negatively correlating with trust in AI assistance [40]. The association might be explained by the fact, that individuals scoring highly in this personality dimension tend to be more self-conscious. Furthermore, we observed a negative correlation between consciousness and performance improvement, which could indicate that more conscientious radiologists did not trust the AI support enough, instead relying on their own skills. This correlation may be confounded by more conscientious individuals already performing better without the model’s assistance. The same holds true for the positive correlation between high extraversion scores and performance improvement, which is likely confounded by the attending radiologists having the lowest scores in *extraversion* and improving least when collaborating with the algorithm. This aligns with a recent study indicating that more accomplished readers profit less from AI assistance than inexperienced ones [38].

AI applications in mammography have been shown to achieve an ROC-AUC of up to 0.95 [1], and human readers achieve a mean sensitivity of 68.9 % and specificity of 88.9 % [41]. The fact that only BI-RADS 4 mammograms were used in our study can explain why neither algorithm nor humans reached these levels (ROC-AUC ResNet50: 0.80, radiologists: 0.75%).

### Limitations

We consider the following limitations of our work: We excluded patients with no detectable mass. As these cases represent a small subgroup of mammography lesions, our findings are not outright generalizable to clinical routine.

Furthermore, as we did not test another algorithm with different accuracy, we cannot evaluate the influence the accuracy of AI has on readers’ decisions. As studies found faulty AI decreasing human performance in collaborative settings [38], further investigation of this aspect is required. Discrepancies between radiologists and the algorithm were not analyzed. To detect systematic failure or to identify cases, in which the algorithm was most useful, further inquiry is needed.

Moreover, the primary effect of the certainty display cannot be inferred as we evaluated it in combination with the heatmap only. As the analyzed types of algorithmic assistance were always presented in the same order, the participating radiologists may have withheld their change of decision based upon the heatmap awaiting the additional information from the prediction and its certainty, which could impose a significant confounding of the results. To eliminate this bias, future studies should vary the order in which radiologists are presented with different types of AI assistance. Our notion of uncertainty is limited because it does not cover out-of-distribution input and does not represent calibrated uncertainty [42]. Techniques based on Bayesian deep learning would have been capable to offer more robust uncertainty estimates [43]. Furthermore, we did not investigate the utilization of visual interpretability methods like Grad-CAM [40] and LIME [7], which have a similar scope.

Also, the communication with the algorithm, or lack of it, is likely to be influenced by radiologists knowing they were being observed and might not reflect their behavior in clinical practice. As all participants evaluated the images first without and later with AI assistance, the recall of images could be an influence on their performance. Lastly, due to the small number of readers, we cannot rule out confounding, especially regarding the influence of personality traits on the readers’ improvement with AI assistance. To overcome these limitations and establish recommendations for improved human-AI interaction, studies with a larger number of participants and different set-ups are required. As the assessment of personality traits and interaction represents an interface to psychological research, the collaboration between these fields of research would be highly beneficial.

## Conclusion

In concordance with previous research, we found the performance of combined human and artificial intelligence superior to both individual groups and that displaying aspects of the classification process of the algorithm to readers leads to increased trust in the algorithm’s performance. This highlights the importance of improved transparency and explainable AI systems. Furthermore, our findings indicate an association between different personality traits and human-AI interaction. Our study differs from previous research investigating different prompt types in CAD applications, as we focused on assessing the impact of different types of AI assistance in addition to object detection. Accordingly, only cropped images simulating the output of object detection algorithms were used in the reader study, which imposes a limitation on our findings. We encourage the validation of our findings in further studies addressing order bias and including different prompt types.

## Supplementary information

ESM 1 (DOCX 740 kb)

## Abbreviations

AI: Artificial intelligence
BI-RADS: Breast Imaging-Reporting and Data System
CAD: Computer-aided diagnosis
ResNet50: 50-layer residual neural network

## Declarations

### Guarantor

The scientific guarantor of this publication is PD Dr. med. Rickmer F. Braren.

### Statistics and biometry

No complex statistical methods were necessary for this paper.

### Methodology

• retrospective

• experimental

• performed at one institution
