# Supplementary material for: Algorithmic transparency and interpretability measures improve radiologists’ performance in BI-RADS 4 classification
Source: Eur Radiol. 2022 Oct 25;33(3):1844–51. doi: 10.1007/s00330-022-09165-9 (PMC9935738; doi:10.1007/s00330-022-09165-9)
Supplement: Supplementary file 1 — (DOCX 740 kb) [file 330_2022_9165_MOESM1_ESM.docx]

A1 - STROBE Checklist

|  | **Item No** | **Recommendation** | Remark/ Location |
| --- | --- | --- | --- |
| Title and abstract | 1 | (*a*) Indicate the study’s design with a commonly used term in the title or the abstract | Abstract (Methods) |
|  |  | (*b*) Provide in the abstract an informative and balanced summary of what was done and what was found | Abstract (Methods, Results) |
| **Introduction** |  |  |  |
| Background/rationale | 2 | Explain the scientific background and rationale for the investigation being reported | Abstract, Introduction |
| Objectives | 3 | State specific objectives, including any prespecified hypotheses | Introduction, Discussion |
| **Methods** |  |  |  |
| Study design | 4 | Present key elements of study design early in the paper | Methods |
| Setting | 5 | Describe the setting, locations, and relevant dates, including periods of recruitment, exposure, follow-up, and data collection | Methods |
| Participants | 6 | (*a*) Give the eligibility criteria, and the sources and methods of selection of participants. Describe methods of follow-up | Methods, Supplement |
|  |  | (*b*) For matched studies, give matching criteria and number of exposed and unexposed | Not applicable |
| Variables | 7 | Clearly define all outcomes, exposures, predictors, potential confounders, and effect modifiers. Give diagnostic criteria, if applicable | Methods, Results, Discussion |
| Data sources/ measurement | 8* | For each variable of interest, give sources of data and details of methods of assessment (measurement). Describe comparability of assessment methods if there is more than one group | Methods |
| Bias | 9 | Describe any efforts to address potential sources of bias | Methods |
| Study size | 10 | Explain how the study size was arrived at | Methods, Supplement |
| Quantitative variables | 11 | Explain how quantitative variables were handled in the analyses. If applicable, describe which groupings were chosen and why | Methods |
| Statistical methods | 12 | (*a*) Describe all statistical methods, including those used to control for confounding | Methods |
|  |  | (*b*) Describe any methods used to examine subgroups and interactions | Methods |
|  |  | (*c*) Explain how missing data were addressed | Not applicable |
|  |  | (*d*) If applicable, explain how loss to follow-up was addressed | Not applicable |
|  |  | (*e*) Describe any sensitivity analyses | Methods, Results |
| **Results** |  |  |  |
| Participants | 13* | (a) Report numbers of individuals at each stage of study—eg numbers potentially eligible, examined for eligibility, confirmed eligible, included in the study, completing follow-up, and analysed | Methods, Supplement |
|  |  | (b) Give reasons for non-participation at each stage | Supplement |
|  |  | (c) Consider use of a flow diagram | Supplement |
| Descriptive data | 14* | (a) Give characteristics of study participants (eg demographic, clinical, social) and information on exposures and potential confounders | Supplement |
|  |  | (b) Indicate number of participants with missing data for each variable of interest | Not applicable |
|  |  | (c) Summarise follow-up time (eg, average and total amount) | Not applicable |
| Outcome data | 15* | Report numbers of outcome events or summary measures over time | Results |
| Main results | 16 | (*a*) Give unadjusted estimates and, if applicable, confounder-adjusted estimates and their precision (eg, 95% confidence interval). Make clear which confounders were adjusted for and why they were included | Results |
|  |  | (*b*) Report category boundaries when continuous variables were categorized | Not applicable |
|  |  | (*c*) If relevant, consider translating estimates of relative risk into absolute risk for a meaningful time period | Not applicable |
| Other analyses | 17 | Report other analyses done—eg analyses of subgroups and interactions, and sensitivity analyses | Results |
| **Discussion** |  |  |  |
| Key results | 18 | Summarise key results with reference to study objectives | Discussion |
| Limitations | 19 | Discuss limitations of the study, taking into account sources of potential bias or imprecision. Discuss both direction and magnitude of any potential bias | Discussion |
| Interpretation | 20 | Give a cautious overall interpretation of results considering objectives, limitations, multiplicity of analyses, results from similar studies, and other relevant evidence | Discussion |
| Generalisability | 21 | Discuss the generalisability (external validity) of the study results | Discussion |
| **Other information** |  |  |  |
| Funding | 22 | Give the source of funding and the role of the funders for the present study and, if applicable, for the original study on which the present article is based | Funding |

*Give information separately for exposed and unexposed groups.

**Note:** An Explanation and Elaboration article discusses each checklist item and gives methodological background and published examples of transparent reporting. The STROBE checklist is best used in conjunction with this article (freely available on the Web sites of PLoS Medicine at http://www.plosmedicine.org/, Annals of Internal Medicine at http://www.annals.org/, and Epidemiology at http://www.epidem.com/). Information on the STROBE Initiative is available at http://www.strobe-statement.org.

A2 - Patient inclusion Flowchart


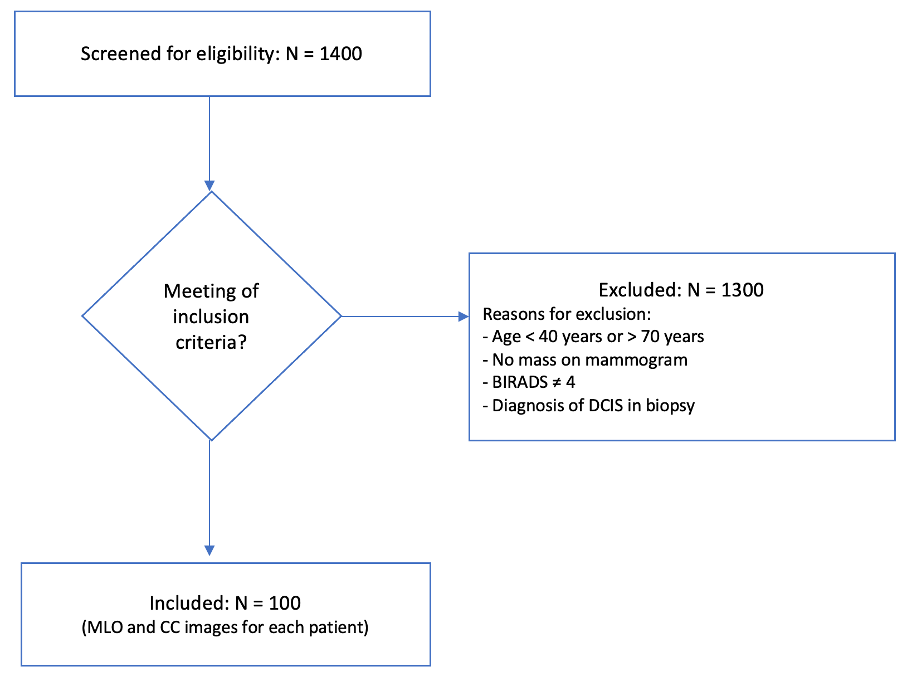


A3 – Questionnaire used in the reader study

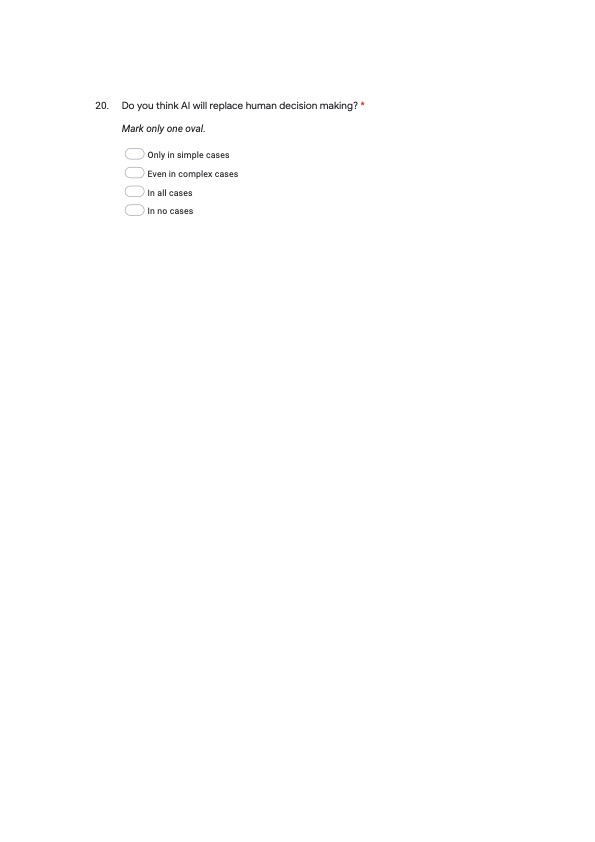


A4 - Big 5 Personality Traits

Definitions and descriptions of the Big Five personality traits based upon [John1999, John2008].

|  | Conceptual definition | Behavioral examples |
| --- | --- | --- |
| Extraversion (Factor I) | Implies an energetic approach toward the social and material world and includes traits such as sociability, activity, assertiveness, and positive emotionality. | Approaches strangers at a party and introduce themselves; Take the lead in organizing a project |
| Agreeableness (Factor II) | Contrasts a prosocial and communal orientation toward others with antagonism and includes traits such as altruism, tender-mindedness, trust, and modesty. | Emphasize the good qualities of other people when they talk about them; Lend things to people they know; Console a friend who is upset |
| Consciousness (Factor III) | Describes socially prescribed impulse control that facilitates task- and goal- directed behavior, such as thinking before acting, delaying gratification, following norms and rules, and planning, organizing, and prioritizing tasks. | Arrive early or on time for appointments; Study hard  in order to get the highest grade in class; Double-check a term paper for typing and spelling errors |
| Neuroticism (Factor IV) | Contrasts emotional stability and even-temperedness with negative emotionality, such as feeling anxious, nervous, sad, and tense. | Get upset when somebody is angry with them; Do not accept the good and the bad in their life without complaining or bragging |
| Openness (Factor V) | Describes the breadth, depth, originality, and complexity of an individual's mental and experiential life. | Take the time to learn something simply for the  joy of learning; Look for stimulating activities that break up my routine |
